# Associations between blood selenium and serum neurofilament light chain: results of a nationwide survey

**DOI:** 10.3389/fneur.2025.1490760

**Published:** 2025-04-08

**Authors:** Yayun Liao, Kejian Zhou, Baoquan Lin, Shan Deng, Lu Qin, Baohui Weng, Hong Yang, Liya Pan

**Affiliations:** Department of Neurology, The Fourth Affiliated Hospital of Guangxi Medical University, Liuzhou, China

**Keywords:** blood Se, sNfL, protective, central nervous system, NHANES

## Abstract

**Background:**

Selenium (Se) is essential for many nervous system functions including memory, cognition and coordination, which has also been linked to a variety of neurological disorders, such as epilepsy, Alzheimer’s disease (AD) and Parkinson’s disease (PD). Serum neurofilament light chain (sNfL) is a biomarker of neurologic diseases. Studies on the relationship between blood Se and sNfL are limited.

**Methods:**

The National Health and Nutrition Examination Survey (NHANES) 2013–2014 data were employed to perform multivariate linear regression analysis and smooth curve fitting in order to investigate the relationship between blood Se and sNfL. Utilizing subgroup analyses and interaction tests, the stability of this relationship between populations was evaluated.

**Results:**

sNfL and blood Se had an inverse relationship in 1,036 individuals who were older than 20. According to the fully adjusted model, the sNfL decreased by 54.75 pg./mL for every unit increase in log blood Se [*β* = −54.75, 95% CI (−75.36, −34.14)]. The sNfL of individuals in the highest blood Se quartile decreased by 3.4 pg./mL in comparison to those in the lowest quartile [*β* = −3.40, 95% CI (−6.47, −0.32)]. This inverse association was more significant in those who were younger than 60 years old, male, normal weight, had a history of smoking and drinking.

**Conclusion:**

Blood Se is inversely associated with sNfL in American adults. Our findings indicate that blood Se may have a potential protective effect against neuronal damage.

## Introduction

Selenium (Se) is a common metal and essential trace element (1) that plays critical roles in immune response, apoptosis, endoplasmic reticulum homeostasis, regulation of transcription factor, development of central nervous system (CNS) and redox homeostasis by selenoproteins (2–4). Se is essential to CNS and involves in a variety of functions, such as memory, cognition, coordination and motor performance (5). Se is associated with the pathology of neurological diseases, including epilepsy, Parkinson’s disease (PD), Alzheimer’s disease (AD), and amyotrophic lateral sclerosis (ALS) (6, 7). Research has shown that low blood selenium level is associated with intractable epilepsy in children. Children with refractory epilepsy have a severe decrease in blood Se (8). Seizures can be controlled when children are supplemented with Se; after withdraw Se, seizures recurs and can only be controlled by re-supplementation with Se (9). Se deficiency is associated with progressive neurological damage and cognitive decline, which is detrimental to AD. In addition, excessive exposure to Se can also cause neurotoxicity. In Florida, an outbreak of selenium poisoning induced by nutritional supplements has been reported, and the disease is related to cognitive and memory and impairment in patients aged 15–57 years (10). Consequently, more research is needed to verify Se′s involvement in neurological disorders in humans.

In central and peripheral nervous system diseases related to axonal injury or degeneration, serum neurofilament light chain (sNfL) concentration is increased, which is a biomarker of nerve axonal injury (11). sNfL is a quantitative indicator of axonal injury, and its elevation may have prognostic value in a variety of neurological diseases. Huntington’s disease (HD), ALS, and traumatic brain injury (TBI) have all been linked to elevated sNfL levels (12–14). However, in multiple sclerosis (MS) and AD, higher sNfL levels are related to worse clinical outcomes and more severe neurological impairment (15, 16).

We collected data on blood Se and sNfL from participants in the National Health and Nutrition Examination Survey (NHANES, 2013–2014) in order to gain a better understanding of the relationship between these two variables. Assessing the relationship between blood Se concentration and sNfL in a US population was the main objective of this study.

## Methods

### Study population

**Figure 1:**
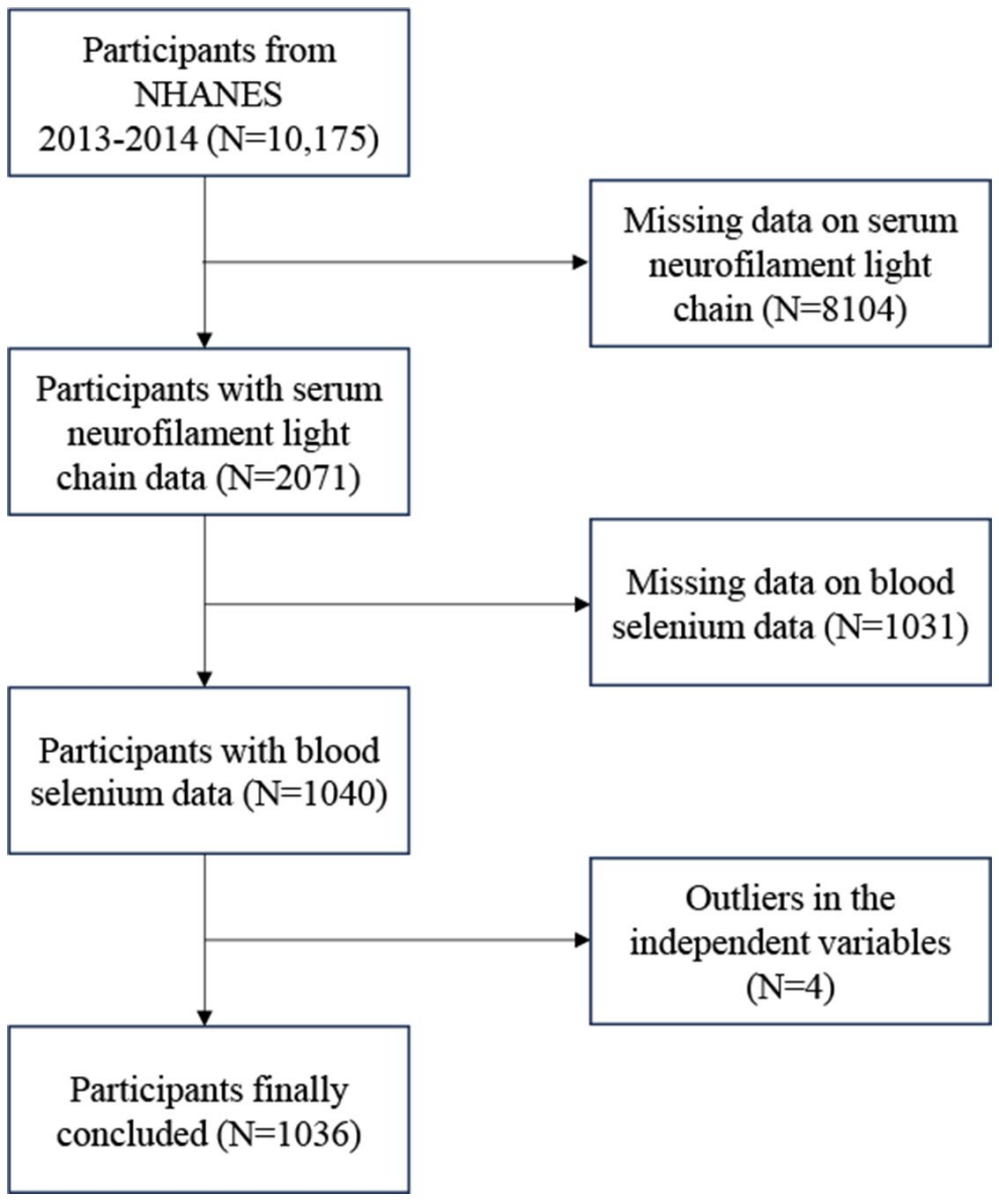
Flow chart of participants selection. NHANES, National Health and Nutrition Examination Survey.

The NHANES is a nationally representative survey conducted in the United States by the Centers for Disease Control and Prevention (17, 18). The findings were authorized by the National Center for Health Statistics’ research ethics review board. Written comments were provided by all participants at recruitment (19, 20). The survey lasted for 2 years (2013–2014), and there was a total survey cycle. We excluded 8,104 participants with no data on sNfL, 1,031 participants with missing data on blood Se, and 4 participants with missing covariates. The study eventually included 1,036 participants (Figure 1).

### Blood Se measurement

Whole blood specimens were collected, processed, stored, and then sent for analysis to the Centers for Disease Control and Prevention and the National Center for Environmental Health. The NHANES laboratory used inductively coupled plasma mass spectrometry to measure blood Se concentrations. The detection limit of blood Se was 24.48 μg/L. Detailed instructions for specimen collection and handling were provided by the NHANES laboratory. With the interquartile range method, the blood Se concentration distribution range was used to divide the blood Se concentration into four quartiles. Quartile is defined as the Q1 (<183.6 μg/L), Q2 (≥183.6 μg/L and < 197.1 μg/L), Q3 (≥197.1 μg/L and ≤211.1 μg/L), and Q4 (>211.1 μg/L).

### sNfL measurement

A highly sensitive immunoassay created by Siemens was used to test serum samples collected between 2013 and 2014 for NfL. A completely automated atelica immunoassay system allowed for the seamless execution of every step. The following links provide additional information and a more thorough protocol: https://wwwn.cdc.gov/Nchs/Nhanes/2013-2014/SSSNFL_H.htm.

### Covariates

Covariates included body mass index (BMI), smoking, alcohol consumption status, age, gender, race, education level, income-to-poverty ratio (PIR), weak kidneys, diabetes, hypertension, and stroke. Demographic data were gathered through participants’ self-reported interviews and included the classification of ethnicity into Mexican American, Non-Hispanic White, Non-Hispanic Black, and Other. The level of education was divided into three tiers: less than high school, high school level, and beyond high school. BMI is calculated by the formula of weight in kilograms divided by the square of height in meters. Smoking was determined by a history of consuming a minimum of 100 cigarettes over a lifetime. Alcohol consumption was defined as consuming more than one drink on average on days during the previous 12 months. With the exception of kidney stones, bladder infections, or urinary incontinence, weak kidneys were defined as having been diagnosed by a physician or other health professional as having weak or failing kidneys. Diabetes in this study was characterized by self-reported diabetes, or glycosylated hemoglobin levels of 6.5% or greater, or fasting blood glucose levels of 126 mg/dL or higher, or the utilization of insulin or hypoglycemic drugs. Hypertension was identified by self-reported hypertension, or systolic blood pressure of 140 mmHg or higher, or diastolic blood pressure of 90 mmHg or higher, or the intake of antihypertensive medications.

### Statistical analyses

The weighted chi-square test and weighted linear regression models were used to assess the participant’s demographics based on blood Se quartile. With weighted multiple linear regression, the linear relationship between blood Se and sNfL was investigated. A trend test was used to examine the linear association trend between blood Se and sNfL after blood Se was converted from a continuous to a categorical (quartile). The relationship between blood Se and sNfL in individuals with varying gender, age, smoking, alcohol intake, BMI, diabetes, and stroke was investigated using subgroup analysis. Additionally, to find out if the associations held true for all subgroups, interaction tests were executed. The nonlinear relationship between sNfL and blood Se was examined through the application of smooth curve fitting. For all analyses, either Empowerstats (version 4.2) or R (version 4.2) were utilized. The statistical significance threshold was set at two-sided p less than 0.05.

## Results

### Baseline characteristics

Among 1,036 participants aged over 20 years, the mean (SD) age was 46.90 (15.16) years, 48.17% were male and 51.83% were female. Baseline characteristics were defined according to blood Se quartiles (Q1, <183.6 μg/L; Q2, ≥183.6 μg/L and <197.1 μg/L; Q3, ≥197.1 μg/L and ≤211.1 μg/L; Q4, >211.1 μg/L). With the increase of blood Se quartile, the proportion of males showed an upward trend, while the proportion of females showed a downward trend (*p* = 0.0018). Compared with participants in the lowest Se quartile, those with higher blood Se was more likely to be overweight. In contrast, participants with low blood Se were more likely to be obese than those with higher blood Se (Table 1).

**Table 1:** Basic characteristics of participants by blood selenium among U.S. adults.

Characteristics	Blood selenium				*p*-value
	Q1 (*n*)	Q2 (*n*)	Q3 (*n*)	Q4 (*n*)	
Age (years)	45.47 ± 14.37 (259)	45.58 ± 15.21 (259)	47.56 ± 15.18 (259)	45.51 ± 15.13 (259)	0.2849
Gender, (%)					0.0018
Male	39.28 (107)	51.53 (121)	55.6 (140)	51.22 (131)	
Female	60.72 (152)	48.47 (138)	44.4 (119)	48.78 (128)	
Race/ethnicity, (%)					0.4229
Non-Hispanic White	61.56 (105)	67.22 (113)	71.42 (122)	67.97 (116)	
Non-Hispanic Black	13.16 (58)	9.2 (45)	7.67 (44)	8.43 (39)	
Mexican American	9.51 (32)	10.97 (42)	8.69 (41)	8.28 (33)	
Other races	15.78 (64)	12.61 (59)	12.21 (52)	15.32 (71)	
Education level, (%)					0.0543
<high school	17.18 (63)	16.34 (57)	12.14 (47)	14.53 (55)	
High school	20.71 (55)	25.07 (59)	18.89 (58)	20.06 (55)	
>high school	62.11 (141)	58.59 (143)	68.97 (154)	65.41 (149)	
Drinking alcohol, (%)					0.3951
Ever	44.28 (114)	45.98 (108)	50.7 (111)	44.31 (110)	
Never	55.72 (145)	54.02 (151)	49.3 (148)	55.69 (149)	
Smoking, (%)					0.8522
Ever	46.67 (125)	42.91 (108)	43.84 (105)	44.57 (115)	
Never	53.33 (134)	57.09 (151)	56.16 (154)	55.43 (144)	
Diabetes, (%)					0.075
Yes	10.78 (35)	14.62 (42)	9.96 (33)	16.68 (54)	
No	89.22 (224)	85.38 (217)	90.04 (226)	83.32 (205)	
Hypertension, (%)					0.0697
Yes	35.98 (99)	35.89 (98)	33.94 (97)	44.51 (114)	
No	64.02 (160)	64.11 (161)	66.06 (162)	55.49 (145)	
Weak kidneys, (%)					0.4435
Yes	4.25 (13)	1.9 (6)	2.79 (11)	2.57 (7)	
No	95.75 (246)	98.1 (253)	97.21 (248)	97.43 (252)	
Stroke, (%)					0.2508
Yes	1.08 (5)	3.06 (5)	1.83 (7)	0.98 (4)	
No	98.92 (254)	96.94 (254)	98.17 (252)	99.02 (255)	
Family PIR	2.82 ± 1.66 (259)	2.93 ± 1.67 (259)	3.04 ± 1.49 (259)	2.91 ± 1.74 (259)	0.5041
BMI, (kg/m^2^)					0.0011
<25	30.76 (82)	28.26 (80)	30.13 (75)	29.63 (78)	
≥25 and <30	26.68 (69)	30.87 (73)	42.69 (100)	35.53 (98)	
≥30	42.56 (108)	40.87 (106)	27.19 (84)	34.83 (83)	

Mean ± SD for continuous variables: the *p*-value was calculated by the weighted linear regression model; (%) for categorical variables: the *p-*value was calculated by the weighted chi-square test. PIR, the ratio of income to poverty; BMI, body mass index; Q, quartile.

### Relationship between blood Se and sNfL

**Figure 2:**
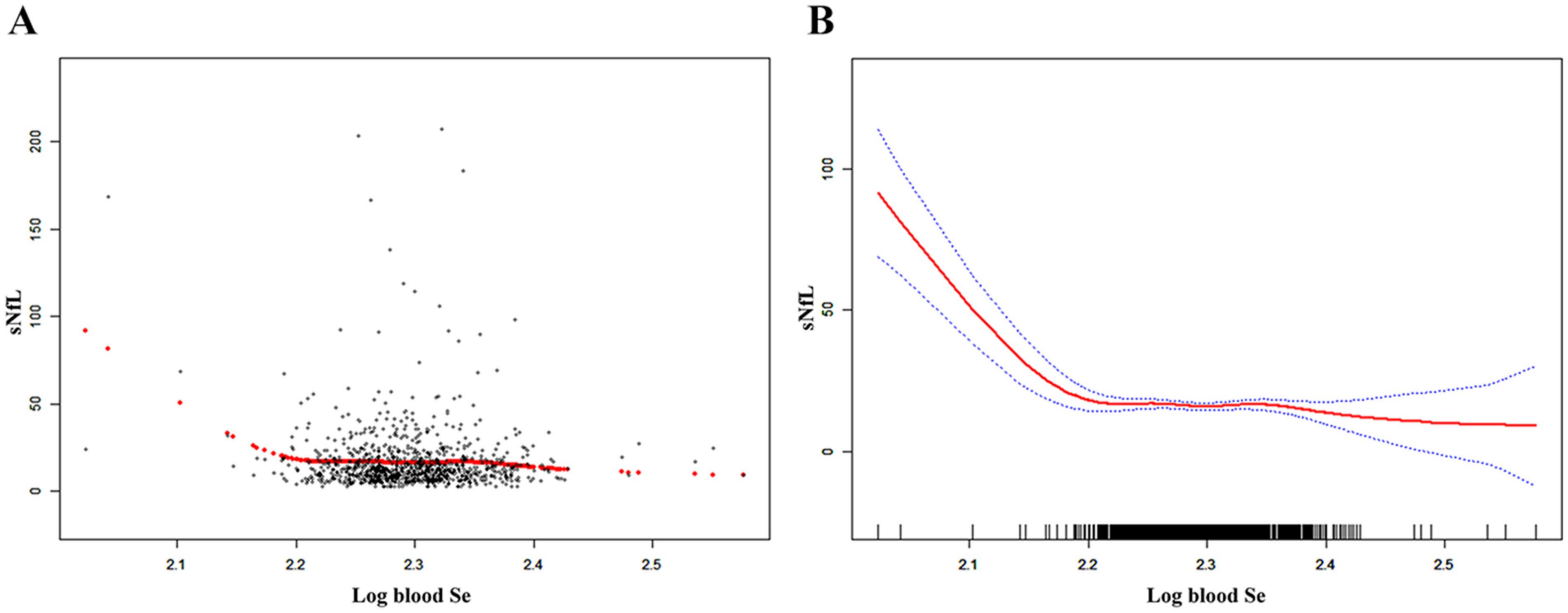
The nonlinear associations between blood Se and sNfL. **(A)** Each black point represents a sample. **(B)** The solid red line represents the smooth curve fit between variables. Blue bands represent the 95% of confidence interval from the fit. Se, selenium; sNfL, serum neurofilament light chain.

Table 2 shows the association between blood Se and sNfL. Because of the sNfL concentration values deviated from the normal distribution, we included the log-transformation of blood selenium variable in the analysis. Log blood Se and sNfL showed a significant negative association in all models: crude [*β* = −50.90, 95% CI (−72.70, −29.10)], partially adjusted [β = −54.72, 95% CI (−75.69, −33.74)], and fully adjusted [β = −54.75, 95% CI (−75.36, −34.14)]. After adjustment for all covariates, sNfL decreased 54.75 pg./mL for each 1-unit increase in log blood Se. Both the fully and partially adjusted models remained statistically significant (all *P* for trend < 0.05) after stratifying log blood Se into quartiles. Compared with the lowest log blood Se quartile group, the sNfL in the highest log blood Se quartile group decreased by 3.4 pg./mL [*β* = −3.40, 95% CI (−6.47, −0.32)]. Additionally, the nonlinear negative association between log blood Se and sNfL was further supported by smooth curve fitting results (Figure 2). Further studies found that the inflection point for the threshold effect was 2.22. As shown in Table 3, before the inflection point, log blood Se and sNfL were negatively associated [*β* = −567.48, 95% CI (−647.77, −487.19)].

**Table 2:** Associations between Log blood Se and sNfL.

Log blood Se	sNfL	*P* for trend
	β (95% CI), *p*-value	
Crude model (Model 1)		
Continuous	−50.90 (−72.70, −29.10), <0.0001	
Categories		0.105
Quartile 1	0 (ref)	
Quartile 2	−2.71 (−5.87, 0.44), 0.0925	
Quartile 3	−3.09 (−6.21, 0.03), 0.0528	
Quartile 4	−2.70 (−5.96, 0.56), 0.1055	
Minimally adjusted model (Model 2)		
Continuous	−54.72 (−75.69, −33.74), <0.0001	
Categories		0.027
Quartile 1	0 (ref)	
Quartile 2	−3.39 (−6.43, −0.35), 0.0291	
Quartile 3	−4.60 (−7.63, −1.57), 0.0030	
Quartile 4	−3.31 (−6.45, −0.17), 0.0390	
Fully adjusted model (Model 3)		
Continuous	−54.75 (−75.36, −34.14), <0.0001	
Categories		0.026
Quartile 1	0 (ref)	
Quartile 2	−2.93 (−5.90, 0.04), 0.0534	
Quartile 3	−3.78 (−6.76, −0.79), 0.0133	
Quartile 4	−3.40 (−6.47, −0.32), 0.0307	

Model 1: no covariates were adjusted. Model 2: age, gender, and race were adjusted. Model 3: age, gender, race, education level, PIR, BMI, drinking alcohol, smoking, hypertension, diabetes, weak kidneys and stroke were adjusted. Se, selenium; sNfL, serum neurofilament light chain; PIR, the ratio of income to poverty; BMI, body mass index; Q, quartile.

**Table 3:** Threshold effect analysis of Log blood Se on sNfL using a two piecewise linear regression model.

sNfL	β (95% CI)	*P*
Log blood Se		
Fitting by standard linear model	−59.26 (−80.15, −38.37)	<0.0001
Fitting by two-piecewise linear model		
Inflection point	2.22	
<2.22	−567.48 (−647.77, −487.19)	<0.0001
>2.22	2.09 (−19.47, 23.66)	0.8493
Log-likelihood ratio	<0.001	

Age, gender, race, education level, PIR, BMI, drinking alcohol, smoking, hypertension, diabetes, weak kidneys and stroke were adjusted. Se, selenium; sNfL, serum neurofilament light chain; PIR, the ratio of income to poverty; BMI, body mass index; Q, quartile.

### Subgroup analyses

We conducted subgroup analysis and interaction tests, stratified by age, gender, BMI, smoking, alcohol consumption, diabetes, and stroke, to ascertain whether the relationship between blood Se and sNfL was consistent in the general population and to identify any potential different population settings (Table 4). Our findings demonstrated that there were significant differences in the association between log blood Se and sNfL in the subgroups of gender, age, BMI, smoking, and drinking. Log blood Se and sNfL were significantly associated negatively in male [*β* = −96.57, 95% CI (−125.79, −67.35)], in individuals under 60 [β = −74.97, 95% CI (−98.74, −51.21)], in smokers [β = −110.62, 95% CI (−140.20, −81.03)], in normal weight participants [β = −124.51, 95% CI (−157.39, −91.63)] and in drinkers [β = −115.41, 95% CI (−146.14, −84.68)]. There was no significant association between log blood Se and sNfL in the remaining patients who were 60 years of age or older, female, overweight, obese, non-smokers and non-drinkers.

**Table 4:** Subgroup analysis of the association between Log blood Se and sNfL.

Subgroup (*n*)	sNfL [β (95%CI)], *p*-value	*P* for interaction
Gender		0.0004
Male (499)	−96.57 (−125.79, −67.35), <0.0001	
Female (537)	−21.32 (−50.79, 8.14), 0.1564	
Age		0.0031
<60 years (774)	−74.97 (−98.74, −51.21), <0.0001	
≥60 years (262)	−0.24 (−44.04, 43.57), 0.9915	
BMI		<0.0001
<25 kg/m^2^ (315)	−124.51 (−157.39, −91.63), <0.0001	
≥25 and < 30 kg/m^2^ (340)	−22.87 (−59.25, 13.51), 0.2182	
≥30 kg/m^2^ (381)	−3.94 (−42.81, 34.93), 0.8425	
Alcohol drinking		<0.0001
Ever (443)	−115.41 (−146.14, −84.68), <0.0001	
Never (593)	−13.33 (−41.13, 14.48), 0.3478	
Smoking		<0.0001
Yes (453)	−110.62 (−140.20, −81.03), <0.0001	
No (583)	−8.65 (−37.49, 20.19), 0.5569	
Diabetes		0.1680
Yes (164)	−18.36 (−80.34, 43.62), 0.5617	
No (872)	−64.27 (−86.33, −42.20), <0.0001	
Stroke		0.0533
Yes (21)	101.92 (−62.73, 266.57), 0.2253	
No (1,015)	−60.25 (−81.01, −39.49), <0.0001	

Age, gender, race, education level, PIR, BMI, drinking alcohol, smoking, hypertension, diabetes, weak kidneys and stroke were adjusted. Se, selenium; sNfL, serum neurofilament light chain; PIR, the ratio of income to poverty; BMI, body mass index; Q, quartile.

## Discussion

We discovered an inverse relationship between blood Se and sNfL in a cross-sectional study involving 1,036 representative participants. These results show that higher blood Se concentration is associated with lower sNfL concentration, suggesting a potential protective effect of Se on neuronal injury. This is the first study to link blood Se to biomarkers of nerve injury in adults. Blood Se concentrations in US adults may also be concerning for neurological health if the correlations we found are causal. However, it is unknown whether sNfL reduction by increased blood Se concentration alleviates neurological disease.

In 1957, selenium was discovered to be a necessary trace element for mammals. Se primarily carries out a range of biological activities via 25 selenoproteins with selenocysteines in the active center. Mammals’ physiological processes, including immune response, thyroid hormone metabolism, antioxidant defense, and fertility, are influenced by selenium (21–23). Selenium is also essential for the brain. According to the study, epileptic patients’ blood Se levels were lower than those of healthy controls (24). Blood Se activity were significantly decreased in children with refractory epilepsy (8), when children with epilepsy are supplemented with Se, seizures can be controlled. In a study of epileptic mice, treatment with Nano-Se reversed PTZ-induced seizure behavior and seizure duration, oxidative damage, and neuronal loss (25). In addition, Se is closely related to cognitive function and movement disorders. A cross-sectional study conducted in rural China showed cognitive decline in 2,000 elderly people aged over 65 years with Se deficiency (26). Hippocampal neuron loss was significantly higher in Se deficient rats than in sufficient rats (27). Furthermore, mice given a diet low in selenium showed changes in hippocampal long-term potentiation, short-term plasticity, and synaptic transmission (28). Blood Se levels were found to be lower in PD patients than in non-PD patients in a cross-sectional study, suggesting that high blood Se levels may protect against PD (29). We discovered an inverse relationship between blood Se and sNfL in the current study, which is consistent with the findings of previously research and suggests that individuals with high blood Se levels may be less likely to have underlying neurological diseases.

The structural proteins that make up the cytoskeleton of nerve cells include neurofilaments, which are made up of light, medium, and heavy chains (30). The amount of NfL released into the extracellular space is correlated with damage to nerve axons. In neurodegenerative diseases that cause damage to neurons, axons break down and release NfL, which eventually finds its way into the peripheral blood and cerebrospinal fluid (31). In clinical practice, sNfL has become a biomarker for a variety of neurological diseases (32). According to a sizable longitudinal cohort study, sNfL levels increased with age and time in PD patients and were associated with the severity of the disease (33). Elevated sNfL levels have been found in neurological diseases such as TBI, HD, and ALS (12–14). However, worse clinical outcomes in multiple sclerosis and AD and more severe neurological impairment are generally associated with higher sNfL levels (15, 16). Furthermore, it was found that hospitalized stroke patients had higher sNfL levels, and there was a positive correlation between sNfL levels and cerebral infarction (34). In individuals with mild ischemic stroke, there is a higher chance of early neurological deterioration when there is an increase in sNfL levels (35), which can also predict adverse clinical outcomes at 90 days after ischemic stroke (36). The results of our subgroup analyses suggest that blood Se does not affect sNfL levels in participants with a history of stroke. However, whether blood Se levels have an effect on subsequent stroke occurrence requires further epidemiological studies.

Serum NfL levels were discovered to have a strong, positive correlation with age. In individuals younger than 35 years, sNfL levels are relatively stable, but begin to increase nonlinearly with age. After controlling for age, gender was also associated with serum NfL levels, with a mean sNfL level of 17.99 pg./mL in males and 15.78 pg./mL in females (37). In our study, we found that the decrease in sNfL was more pronounced in men and in participants younger than 60 years of age with increasing blood Se levels. It is well known that obesity, smoking, and alcohol consumption are risk factors for vascular events. In a study of the relationship between morbid obesity and circulating NfL, absolute circulating NfL concentrations were found to be lower in morbidly obese subjects than in lean individual participants (38). According to Zhu et al. (39), there is a strong positive correlation between sNfL levels and serum cotinine, which raises the possibility that smoking is a contributing factor to neurological impairment. The high level of sNfL in alcohol dependent individuals suggests that sNfL can be used as a biomarker to evaluate alcohol-induced brain damage (40). Our results showed that sNfL levels were significantly decreased in participants with a history of smoking and drinking as the blood Se concentration increased. Selenium may have a protective effect on nervous system injury induced by smoking and drinking.

It is important to note that excessive Se exposure can also cause neurotoxicity, including memory and cognitive impairment. High doses of Se induce prooxidative effects and toxicity (41). Zeng et al. (29) found a nonlinear connection between blood selenium levels and PD, with high selenium levels significantly lowering the probability of PD development compared to low selenium levels. Around 2.4 μmol/L was the inflection point, after which the rate of risk reduction dramatically dropped as selenium levels rose. Consequently, we carried out a threshold effect analysis, and the findings showed that the inflection point was present at 2.22. When the Log blood Se exceeded 2.22, the sNfL level maintained a relatively stable level in the population. It is suggested that the neuroprotective effect is diminished and even neurotoxicity may occur when blood selenium levels surpass a particular threshold. This study provides a basis for dietary Se intake and maintenance of blood Se level in the American population in the future.

There are certain restrictions on our study. Firstly, the design of the cross-sectional analysis prevented us from establishing a causal relationship between blood Se and sNfL. Longitudinal studies would be necessary to confirm the link. Second, it was not possible to screen out all patients with neurological diseases related to axonal injury or subclinical neurological diseases. Finally, this study only collected blood samples from participants at a single time point and could not dynamically analyze changes in sNfL.

## Conclusion

In conclusion, our study showed that a negative association between blood Se and sNfL, which is affected by gender, age, BMI, smoking, and drinking. When the blood Se concentration reaches a certain level, the sNfL level in the population is maintained at a relatively stable level even with the increase of blood Se concentration.

## Data Availability

The original contributions presented in the study are included in the article/supplementary material, further inquiries can be directed to the corresponding author.
